# Crystallization and preliminary X-ray crystallographic analysis of latent isoform PPO4 mushroom (*Agaricus bisporus)* tyrosinase

**DOI:** 10.1107/S2053230X14000582

**Published:** 2014-01-23

**Authors:** Stephan Gerhard Mauracher, Christian Molitor, Rami Al-Oweini, Ulrich Kortz, Annette Rompel

**Affiliations:** a Institut für Biophysikalische Chemie, Universität Wien, Althanstrasse 14, 1090 Wien, Austria; b School of Engineering and Science, Jacobs University, PO Box 750 561, 28759 Bremen, Germany

**Keywords:** tyrosinases, zymogens, polyphenol oxidase 4, *Agaricus bisporus*, polyoxometalates

## Abstract

Tyrosinase exhibits catalytic activity for the *ortho*-hydroxylation of monophenols to diphenols as well as their subsequent oxidation to quinones. Owing to polymerization of these quinones, brown-coloured high-molecular-weight compounds called melanins are generated. The latent precursor form of polyphenol oxidase 4, one of the six tyrosinase isoforms from *Agaricus bisporus*, was purified to homogeneity and crystallized. The obtained crystals belonged to space group *C*121 (two molecules per asymmetric unit) and diffracted to 2.78 Å resolution. The protein only formed crystals under low-salt conditions using the 6-tungstotellurate(VI) salt Na_6_[TeW_6_O_24_]·22H_2_O as a co-crystallization agent.

## Abstract 2

Polyphenol oxidase 4 (PPO4) from the natural source *A. bisporus* was crystallized in its latent precursor form (pro-tyrosinase; Ser2–Thr565) using the 6-tungstotellurate(VI) salt Na_6_[TeW_6_O_24_]·22H_2_O as a crystallization additive.

## 1. Introduction   

Tyrosinases (EC 1.14.18.1 and 1.10.3.1) are type 3 copper enzymes that are widely distributed in nature and catalyze the two reactions inducing the formation of brown-coloured compounds called melanins (Sánchez-Ferrer *et al.*, 1995 ▶). These reactions, the *ortho*-hydroxylation of monophenols and their subsequent oxidation to quinones, are of interest since the impairment and ageing-related browning of agricultural products represent a concern in the food industry. Additionally, the impact of the generated pigments as stress-resistance and immune-defence factors is a current area of research (Bell & Wheeler, 1986 ▶; Jacobson, 2000 ▶). Similar to other polyphenol oxidases (PPOs), tyrosinases are known to be expressed as inactive zymogens which are activated by the proteolytic removal of the C-­terminal active-site-shielding domain of the enzyme (Yamaguchi *et al.*, 1970 ▶). This maturation process as well as the supplementary functions of the C-terminal domain, *e.g.* copper incorporation, are currently an area of tyrosinase research (Faccio *et al.*, 2013 ▶). Recently, the published crystal structure of a recombinantly expressed pro-tyrosinase from *Aspergillus oryzae* (*ao*TYR; Fujieda *et al.*, 2013 ▶) demonstrated that the C-terminal domain plays a crucial role in the incorporation of copper ions into the active site.

**Figure 1:**
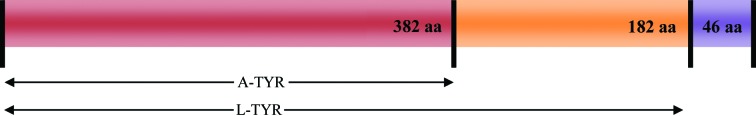
Schematic illustration of the polypeptide chain of PPO4 mushroom tyrosinase. The polypeptide chain of active tyrosinase (core region) is coloured red. The C-terminal domain is coloured orange. The missing C-terminal tail is coloured purple. Aa, amino acids; A-TYR, active tyrosinase; L-TYR, latent tyrosinase.

In the white edible mushroom (*Agaricus bisporus*), six gene sequences (*ab*PPO1–6) encoding tyrosinases are present (Wichers *et al.*, 2003 ▶; Wu *et al.*, 2010 ▶; Weijn *et al.*, 2013 ▶; http://genome.jgi-psf.org/pages/search-for-genes.jsf?organism=Agabi_varbisH97_2). Although expression studies have shown that different isoforms are expressed in differing quantities depending on the growth stage and the tissue type (fruit-body compartment, mycelia *etc.*), no significant assignment with respect to functionality or responsibility could be established for any of these six isoforms (Weijn *et al.*, 2013 ▶). However, the two by far most abundantly expressed isoforms are *ab*PPO3 (UniProt C7FF04) and *ab*PPO4 (UniProt C7FF05). The crystal structure of *ab*PPO3 showed that the active form of the enzyme exhibits a hetero-tetrameric protein conformation (H_2_L_2_) involving a small subunit to which no functionality has yet been assigned (Ismaya *et al.*, 2011 ▶). Recently, a biochemical study (Mauracher *et al.*, 2014 ▶) comprehensively characterized *ab*PPO4 in its zymogen (latent) form (Fig. 1 ▶). The protein was purified in its latent state, lacking only a small transmembrane anchor-containing C-terminal domain (46 amino acids). Several post-translational modifications as well as multiple strain-related mutations were additionally detected. *ab*PPO4 has the highest sequence identity to *ab*PPO3 among structurally known proteins (sequence identity 52%, solely active form); however, only structural knowledge of the C-terminal domain of *ao*TYR, which has very low sequence identity (11%), is presently available.

Polyoxometalates (POMs) are discrete metal–oxo cluster anions of early transition metals in high oxidation states, exhibiting a unique structural and compositional variety (Pope, 1983 ▶; Pope & Kortz, 2012 ▶). POMs are potentially useful in many different areas owing to their thermal, redox, magnetic, optical and bioactive properties (Müller *et al.*, 2001 ▶; Schnack *et al.*, 2006 ▶; Kortz *et al.*, 2009 ▶; Iqbal *et al.*, 2013 ▶; Jahier *et al.*, 2013 ▶). The shape, size and negative charge of POMs allow their binding to positively charged regions of proteins (Zhang *et al.*, 2007 ▶). The use of POMs in protein crystallography has mostly been limited to protein crystal soaking, trying to utilize the POMS for phasing either by isomorphous replacement or anomalous scattering (Corey *et al.*, 1962 ▶; Ladenstein *et al.*, 1987 ▶; O’Halloran *et al.*, 1987 ▶; Thygesen *et al.*, 1996 ▶; Rudenko *et al.*, 2003 ▶; Zebisch *et al.*, 2012 ▶; Dahms *et al.*, 2013 ▶) as well as using POMs as ‘contrast agents’ to display buried channels within protein structures (Dauter, 2005 ▶). Noteworthy is the famous crystal structure of ribosome D50S (*Deinococcus radiodurans*), where crystals were soaked with the Keggin-type POM salt K_5_H[PW_12_O_40_]·12H_2_O (Schluenzen *et al.*, 2000 ▶; Harms *et al.*, 2001 ▶; Pioletti *et al.*, 2001 ▶).

In this work, the crystallization of *ab*PPO4, purified by the method of Mauracher *et al.* (2014 ▶), with the 6-tungstotellurate(VI) salt Na_6_[TeW_6_O_24_]·22H_2_O as a co-crystallization agent is presented.

## 2. Materials and methods   

### 2.1. Sample preparation   

The protein was isolated and purified by the method described by Mauracher *et al.* (2014 ▶). Extraction of the enzyme from the natural source (mushrooms) with prevention of protein-interfering reactions (amino-acid oxidation, protein aggregation, protein cross-linking *etc.*) was established using a distinctly developed method that relies on detergent and soluble polymer (PEG) phase separations. The latent enzyme was kept in its precursor state by the extended usage of protease-inhibition agents and was purified to homogeneity by fast protein liquid chromatography (FPLC) using several ion-exchange columns. Purity was checked by SDS–PAGE and nanoESI–qTOF. For crystallization experiments, the protein was kept at a concentration of 10 mg ml^−1^ in 10 m*M* HEPES buffer pH 7.5.

### 2.2. Synthesis of Na_6_[TeW_6_O_24_]·22H_2_O   

The hydrated sodium salt of hexatungstotellurate(VI) was synthesized according to a modified procedure (Roy & Mishra, 1978 ▶; Schmidt *et al.*, 1986 ▶). Na_2_WO_4_·2H_2_O (5.0 g, 15.2 mmol) and Te(OH)_6_ (0.6 g, 2.6 mmol) were first dissolved in 100 ml water. The pH of the mixture was then adjusted to 5.0 using aqueous HCl solution (1 *M*), which was followed by heating at 383 K until three-quarters of the volume remained. The mixture was then cooled to room temperature and filtered. Slow evaporation of the filtrate at room temperature led to the formation of colourless crystals within one week. The crystals were collected and air-dried and their identity was confirmed by infrared spectroscopy in the solid state.

### 2.3. Protein crystallization   

Initial crystallization screens were performed by the sitting-drop vapour-diffusion technique (96-well Crystal Quick plates, Greiner Bio-One) using a nanodispenser robot (Gryphon, Art Robbins). Screening over a broad variety of crystallization conditions (Crystallization Basic Kit for Membrane Proteins, Crystallization Low Ionic Strength Kit for Proteins and Crystallization Basic Kit for Proteins from Sigma–Aldrich; Pi-PEG Screen HTS, JBScreen Classic 1–10 and JBScreen Membrane 1–3 from Jena Bioscience) gave the first hits (microcrystalline precipitant) for crystallization.

**Figure 2:**
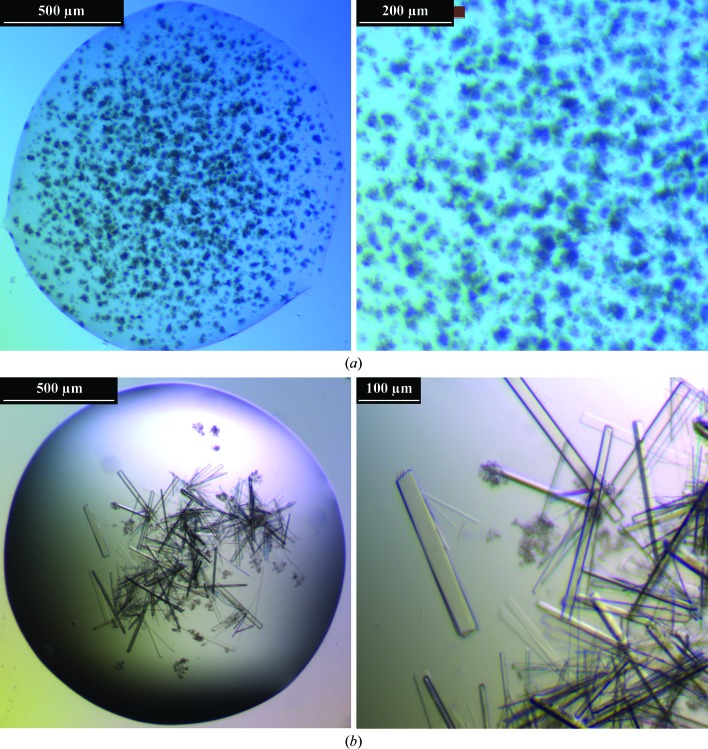
Crystal images of PPO4 mushroom tyrosinase (left, total drop image; right, enlarged image). (*a*) Wispy microcrystals (sea urchins) obtained using MgCl_2_ as a crystallization additive (10% PEG 4000, 15 m*M* MgCl_2_, 25 m*M* Tris–HCl pH 7.5). (*b*) Flat rod-shaped crystals obtained using the POM as a crystallization additive [10% PEG 4000, 1 m*M* Na_6_[TeW_6_O_24_]·22H_2_O, 25 m*M* Tris–HCl pH 7.5].

The first microcrystals were obtained by the hanging-drop vapour-diffusion technique (15-well EasyXtal 10 × 15 plates, Qiagen) using 10% PEG 4000, 15 m*M* MgCl_2_, 25 m*M* Tris–HCl pH 7.5 at 291 K as the crystallization condition (Fig. 2 ▶ *a*). The final crystallization condition for the growth of single crystals suitable for diffraction measurements was 10% PEG 4000, 1 m*M* Na_6_[TeW_6_O_24_]·22H_2_O, 25 m*M* Tris–HCl pH 7.5 at 291 K using 1 µl protein solution (10 mg ml^−1^) and 0.5 µl reservoir solution in the hanging drop and 500 µl solution in the reservoir (Fig. 2 ▶ *b*). The first crystals appeared after 1–2 d and crystal growth came to a stop after approximately 5 d.

### 2.4. Data collection and processing   

Single crystals were harvested by transferring them with a cryoloop (10 µm, 0.1–0.2 mm; Hampton Research) into a 1 µl drop of cryoprotectant solution [20% PEG 4000, 25% PEG 400, 1 m*M* Na_6_[TeW_6_O_24_]·22H_2_O, 25 m*M* Tris–HCl pH 7.5] and were subsequently flash-cooled in liquid nitrogen. The diffraction of about 15 crystals of suitable size was measured at Diamond Light Source, Oxfordshire, England on the monochromatic (0.9173 Å) MX beamline I04-1 equipped with a PILATUS 2M detector. Data sets were collected at 100 K with an oscillation range of 0.5° and an exposure time of 0.5 s. The best crystal diffracted to 2.78 Å resolution using a crystal-to-detector distance of 300.9 mm. The obtained diffraction data sets were processed using the *XDS* program package (version March 30, 2013; Kabsch, 2010 ▶). The space group was determined using the program *POINTLESS* from the *CCP*4 program suite (v.6.3.0; Winn *et al.*, 2011 ▶).

## 3. Results and discussion   

By applying the method described by Mauracher *et al.* (2014 ▶), the obtained protein was purified to homogeneity; hence, any other non-target proteins or different isoforms (*e.g.* PPO3) could be removed entirely (SDS–PAGE, nanoESI–qTOF). However, the preparation showed a somewhat proteolytically ragged C-terminus such that five differing species possessing alternate C-terminal amino acids (polypeptide backbone Ser2–Gly563/Thr565/Gly568/Ala569/Thr570) occur. The species ending with Thr565 was the most abundant, at a ratio of about 6:1 (as determined by nanoESI–qTOF; Mauracher *et al.*, 2014 ▶).

Initial attempts to crystallize the protein covering a wide range of crystallization conditions proved to be unsuccessful with regard to obtaining single crystals; however, a microcrystalline precipitate was obtained using 10% PEG 4000 in the pH range 7–8.5. The best results could be achieved by using magnesium chloride as an additive at rather low concentrations (5–20 m*M*). Low salt concentrations in a basic pH range appeared to be crucial in order to obtain wispy sea-urchin-like microcrystals (Fig. 2 ▶ *a*). However, alteration of any of the crystallization parameters (pH, precipitation agent, temperature or additives), either qualitatively and/or quantitatively, did not help to obtain single crystals. By substituting the magnesium chloride with the 6-tungstotellurate(VI) salt Na_6_[TeW_6_O_24_]·22H_2_O, crystals suitable for X-ray diffraction experiments were obtained (in 1–5 d). The functional concentration range of this POM under these conditions was 0.5–3 m*M*, with a clear optimum at 1 m*M*. In Fig. 2 ▶, the difference between the use of MgCl_2_ and POM as an additive under otherwise identical conditions is presented. The occurrence of multiple, rod-shaped, closely clustered and intergrown crystals was still not preventable, but some single crystals became detached. Such crystals of reasonable size (300 × 30 × 10 µm) were used for X-ray diffraction analysis (Fig. 2 ▶ *b*).

Statistics for the X-ray diffraction measurements are given in Table 1 ▶. The crystals belonged to space group *C*121, with unit-cell parameters *a* = 213.57, *b* = 83.73, *c* = 66.95 Å, β = 102.522°, and diffracted to a maximum resolution of 2.78 Å. Considering the precisely known molecular weight of 64.24 kDa per monomer and assuming the presence of two monomers (as proposed by *phenix.xtriage* from the *PHENIX* program suite; Adams *et al.*, 2010 ▶) per asymmetric unit gives a Matthews coefficient (Matthews, 1968 ▶) of ∼2.29 Å^3^ Da^−1^ and a solvent content of 46%.

We are currently attempting to solve the crystal structure by using molecular replacement and single-wavelength anomalous dispersion (MR–SAD; using *AutoSol* from the *PHENIX* program suite) provided by the POM (tungsten signal), respectively. Several search models for MR are available: *A. bisporus* PPO3 (sequence identity 52%, solely active form; Ismaya *et al.*, 2011 ▶), *Aspergillus oryzae* tyrosinase; sequence identity 21%, full-length pro-enzyme; Fujieda *et al.*, 2013 ▶) and *Octopus dofleini* haemocyanin (sequence identity 16% for copper-binding domain; Cuff *et al.*, 1998 ▶).

## Figures and Tables

**Table 1:** Data-collection and processing statistics for *A. bisporus* tyrosinase (PPO4) crystals Values in parentheses are for the outermost resolution shell.

Space group	*C*121
Wavelength (Å)	0.9173
No. of images	400
Oscillation (°)	0.5
Resolution range (Å)	48.14–2.784 (2.884–2.784)
Completeness (%)	95.89 (96.04)
*R*_merge_ †	0.1657 (0.8243)
〈*I*/σ(*I*)〉	8.70 (1.87)
Multiplicity	4.0 (4.0)
Unit-cell parameters (Å, °)	*a* = 213.57, *b* = 83.73, *c* = 66.95, β = 102.522
*R*_p.i.m._ ‡	0.093 (0.466)
CC_1/2_	0.987 (0.62)
No. of reflections collected	110549 (11068)
No. of unique reflections	27846 (2762)

† *R*_merge_ = 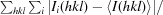 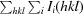.

‡ *R*_p.i.m._ = 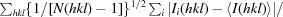 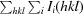, where *I_i_*(*hkl*) is the *i*th observation of reflection *hkl* and 〈*I*(*hkl*)〉 is the weighted average intensity for all observations of reflection *hkl*.
